# Genotype Mutations in Egyptian Children with Familial Mediterranean Fever: Clinical Profile, and Response to Colchicine

**DOI:** 10.31138/mjr.31.2.206

**Published:** 2020-06-15

**Authors:** Hala S. Talaat, Maha F. Sheba, Rehab H. Mohammed, Mohamed Ali Gomaa, Nihal El Rifaei, Mohamed Farouk M. Ibrahim

**Affiliations:** 1 Department of Pediatrics, Faculty of Medicine, Cairo University, Egypt; 2 Ministry of Health, Cairo, Egypt

**Keywords:** Familial Mediterranean fever, MEFV, colchicine

## Abstract

**Background:**

Familial Mediterranean fever (FMF) is an autosomal recessive autoinflammatory disorder that is characterized by recurrent episodes of fever, peritonitis, pleuritis, pericarditis, and/or arthritis. MEFV is the responsible gene for FMF, of which more than 310 mutations have been reported; M694V, M694I, V726A, E148Q, and M680I mutations are the five most frequent mutations responsible for the majority of FMF patients in the Middle East.

**Aim:**

To study the genetic background of FMF among Egyptian children to detect the most frequent MEFV mutations and to study the response of colchicine therapy with different gene mutations.

**Methods:**

This cross-sectional study included 109 pediatric patients already diagnosed clinically with FMF, and were following-up at the Rheumatology Outpatient Clinic, Children’s Hospital, Cairo University.

**Results:**

Out of 109 patients, 95 had positive-MEFV mutation (87.16%), of which the most frequent mutations were E148Q (24/95 patients, 25.26%), V726A (19/95 patients, 20%), M680I (19/95 patients, 20%), M694V (17/95 patients, 17.89%), and M694I (7 patients, 7.37%). A better response to colchicine therapy was noted in E148Q mutation; on the other hand, more severe cases were reported with M694V mutations.

**Conclusion:**

E148Q, V726A, M680I, M694V and M694I mutations are the most frequent mutations denoting the heterogeneous mutation pattern and the milder form of the disease among Egyptian patients. M694V mutations may indicate a more severe disease score.

Familial Mediterranean fever (FMF) is the most common monogenic auto-inflammatory with a high prevalence among people living around the Mediterranean basin such as Jews, Turks, Armenians, and Arabs ^1,2^ FMF has an autosomal recessive pattern of inheritance; however, a significant proportion of patients with only one mutation (ie, heterozygotes) were clinically diagnosed with FMF and responded well to therapy.^3^ FMF is characterized by recurrent episodes of fever along with various clinical manifestations due to inflammation in the peritoneum, synovium, and pleura; amyloidosis and renal failure may develop as a complication of the disease.^4^ Molecular study is important for establishing the diagnosis in patients with clinical criteria of FMF; MEFV, the responsible gene (from *Me*diterranean *F*e*V*er), is mapped on chromosome 16 p13.3, consists of 10 exons and encodes for a 781 amino acid protein called Pyrin, expressed in granulocytes, and is considered a negative regulator of inflammation.^5^ About 310 sequence variants of the MEFV gene had been identified; however, not all variants are associated with the disease phenotype.^6^ The five most common MEFV mutations responsible for the majority of FMF patients in the Middle East are M694V, M694I, V726A, E148Q, and M680I.^7^ Colchicine is the standard first line therapy, and concentrates primarily in neutrophils, inhibiting chemotactic activity during attacks.^8^ The disease severity and response to therapy vary with the different genotypes; homozygous M694V mutation may experience a more severe disease course and had been reported with a relative resistance to colchicine.^9^ The aim of this study was to determine the genetic background of FMF, to detect the most frequent MEFV mutations among Egyptian children, and to assess the clinical profile and the response to colchicine therapy with different genotypes.

## PATIENTS AND METHODS

This cross-sectional study included 109 children with established diagnosis of FMF clinically and according to gene mutations, during their routine regular follow-up at the Paediatric Rheumatology Clinic, Children’s Hospital - Cairo University, in order to detect the most frequent MEFV mutations among our FMF patients, and to assess the clinical profile and the response to colchicine therapy with different genotypes. Diagnosis of FMF was done according to Tel Hashomer criteria.^10^ Tel Hashomer criteria for FMF include: 1) *major criteria*: a) recurrent febrile episodes accompanied by serositis, b) amyloidosis of AA-type without a predisposing disease, c) favourable response to continuous colchicine treatment, and 2) *minor criteria*: a) recurrent febrile episodes, b) Erysipelas-like erythema (ELE), c) FMF in a first-degree relative. FMF can be diagnosed if two major criteria or one major criterion and two minors exist. However, if only one major and one minor criterion exists, the diagnosis is only probable.

A detailed history and thorough physical examination were performed in all patients, their medical records reviewed and a questionnaire given to their parents/caregivers, focusing on: demographic data, family history of FMF, consanguinity, age of onset, time interval between onset and diagnosis of the disease, clinical manifestations, the number of attacks per year, and colchicine therapy regarding the dose to control attacks and response to therapy: patients were either attack-free, had incomplete response (decline >50% in frequency of attacks), or unresponsive to therapy. Severity of the disease was calculated according to Tel Hashomer Severity Score.^11^ Total points indicate: mild disease (2–5 points); moderate disease (6–10 points); severe disease (>10 points).

Results of MEFV mutation analysis were reported. Accordingly, our patients were either homozygotes (had gene mutations on both alleles), heterozygotes (had gene mutation on one allele only), or compound heterozygotes (had two different mutant alleles). Negative-MEFV patients are those patients in whom gene mutations could not be detected. Genomic DNA was extracted by taking 5 ml of patient’s whole blood using the conventional phenol-chloroform extraction method, and then using a reverse hybridization, test strip-based assay (FMF StripAssay; ViennaLab Labordiagnostika, Vienna, Austria) that allows detection of the 12 most frequent MEFV mutations; these are: E148Q, P369S, F479L, M680I, M680I, I692del M694V, M694I, K695R, V726A, A744S, and R761H.

Research Committee of the Department of Pediatrics, Cairo University approved the study. The researchers introduced themselves to parents/caretakers of patients; clear explanations about the aim and nature of the study were discussed by the researchers, and consents were obtained from them. Gene study was done routinely for children with clinically suspected FMF to confirm the diagnosis.

Data were described in terms of mean±SD, median and range, or frequencies and percentages when appropriate. For comparing numerical variables, Mann-Whitney U-test for independent samples was used, and for comparing categorical data, Chi square test was done. P-value <0.05 was considered a statistically significant. All statistical calculations were done using computer programs SPSS (Statistical Package for the Social Science; SPSS Inc., Chicago, IL, USA) version 16 for Microsoft Windows.

## RESULTS

According to gene mutations and allele status, our patients were classified into: Positive MEFV (95 patients, 87.16%) and were either homozygous, heterozygous, or compound heterozygous, and negative-MEFV (n=14, 12.84%). Here we focused on positive-MEFV patients. Out of 95 (positive-MEFV) FMF patients, 46 were males (48.42%) and 49 were females (51.58%). Mean age of onset of FMF was 4.67±2.43 years (range 0.6–10, median 4 years), mean age at diagnosis was 6.94±2.88 years (range 1–13, median 7 years), and interval between onset and diagnosis was 2.36±1.87 years (range 0.5–9, median 2 years). Family history of FMF was reported in 21 (22.11%), consanguinity was detected in 36 patients (37.89%).

Homozygous state was detected in 24/95 patients (25.26%), while heterozygous state was detected in 63/95 patients (66.32%), and compound heterozygotes in 8/95 patients (8.42%). Significant differences regarding number of cases with positive family history of FMF (p-value=0.01), and lower age at diagnosis (p-value=0.03) were noted in homozygotes, 5.86±2.85 years (median 5, range 0.5–12) than heterozygotes/compound heterozygotes, 7.30±2.81 years (median 8, range 1–13). Parental consanguinity was reported in a higher percentage among homozygotes; however, that was not significant (*Table 1*).

**Table 1.** Comparison between homozygotes, and heterozygotes/compound heterozygotes positive-MEFV regarding demographic data.

**Variable**	**Homozygote (n=24)**	**Heterozygote/compound heterozygote**			P-value^*^
		**Heterozygote (n=63)**	**Compound (n=8)**	**Total (n=71)**	
Male/female	14/10	29/34	3/5	32/39	0.26
Consanguinity, n (%)	13 (54.16%)	20 (31.75%)	3 (37.5%)	23 (32.39%)	0.06
Family history of FMF, n (%)	10 (41.67%)	10 (15.87%)	1 (12.5%)	11 (15.49%)	0.01^*^
Disease-associated, n (%)	3 (12.5%)	9 (14.26%)	1 (12.5%)	10 (14.08%)	0.85
Age of onset mean±SD (median, range) years	4.10±2.49 (median 3.5, range 1–9.6)	5.05±2.31 (median 5)	3.33±2.67 (median 3)	4.86±2.40 (median 5, range 1–10)	0.19
Interval between onset and diagnosis mean±SD (median, range) years	2.10±1.52 (median 2, range 0.5–7)	2.35±1.76 (median 2)	3.39±3.18 (median 3)	2.47±1.97 (median 2, range 0.5–10)	0.40
Age at diagnosis mean±SD (median, range) years	5.86±2.85 (median 5, range 0.5–12)	7.38±2.58 (median 8)	6.69±4.42 (median 6.5)	7.30±2.81 (median 8, range 1–13)	0.03^*^

* *P*-value less than 0.05 is considered statistically significant. P-value refers to the comparison between homozygotes and heterozygotes/compound heterozygotes

Results of *MEFV* gene mutations analysis revealed that the most frequent mutations detected in our patients were E148Q mutation in 24/95 patients (25.26%), V726A mutation in 19/95 patients (20%), M680I mutation in 19/95 patients (20%), M694V mutation in 17/95 patients (17.89%), and M694I mutation in 7/95 patients (7.37%) (*Table 2*).

**Table 2.** Gene mutations and Alleles analysis in FMF patients.

**Mutations**	**Patients (n=95)**		
	**Genotype**	**No.**	**%**
Homozygous (n=24, 25.26%)	E148Q/E148Q	3	3.16
	V726A/V726A	2	2.11
	M680I/M680I	5	5.26
	M694V/M694V	9	9.47
	M694I/M694I	5	5.26
Heterozygous (n=63, 66.32%)	E148Q	21	22.11
	V726A	17	17.89
	M680I	14	14.74
	M694V	8	8.42
	M694I	2	2.11
	P369S	1	1.05
Compound heterozygous (n=8, 8.42%)	M680I-V726A	2	2.11
	M694I-E148Q	1	1.05
	M694V-V726A	1	1.05
	M680I-P369S	1	1.05
	M680I-A744S	1	1.05
	M694V-M694I	1	1.05
	M762V-M694V	1	1.05

Fever was the most common manifestation and was reported in 92/95 patients (96.84%), followed by abdominal pain in 90/95 patients (94.74%), and arthritis 75/95 patients (78.95%), ELE in 19/95 patients (20%), and chest pain in 17/95 patients (17.89%). Proteinuria was reported in 5/95 patients (5.26%) in whom renal biopsy was performed; E148Q (2 patients), M694V (2 patients), and M680I-P369S mutations were noted. None of our patients developed amyloidosis, however, family history of amyloidosis was reported in 4 patients (4.21%); M694V heterozygous, E148Q heterozygous, M694V-V726A, and M680I-A744S mutations.

FMF was associated with other disorders in 13 patients (13.68%); M680I was the most frequently associated mutation, and was detected in 5/95 patients (5.26%), followed by E148Q mutation in 3/95 patients (3.16%), V726A and M694V mutations in 2/95 patients each (2.11%), as follows:

- Juvenile idiopathic arthritis (JIA) in 4/95 patients (4.21%), associated with M680I (2 patients), E148Q, and M694V-V726A mutations.
- Henoch-Schoenlein purpura (HSP) in 3/95 patients (3.16%), associated with M680I, E148Q, and M694V mutations.
- Systemic lupus erythematosus (SLE) in 3 patients (3.16%), associated with M680I, E148Q, and V726A mutations.
- Inflammatory bowel disease (IBD) in 2 patients (2.11%), associated with V726A and M694V mutations.
- Both SLE and IBD in one patient (1.05%), associated M680I mutation.

Our patients were receiving colchicine therapy; with a mean dose of 1.38±0.41 mg/day, response to therapy was evaluated. A significantly higher mean dose to control attacks (1.53±0.35 mg/day) was required for homozygotes as compared to heterozygotes/compound heterozygotes (1.33±0.42 mg/day) (p-value=0.04) with a mean dose 1.34±0.42 mg/day for heterozygous, and 1.25±0.46 mg/day for compound heterozygous. The mean number of attacks was significantly reduced from 32.76±17.51 (range 10–96, median 24) per year, before therapy, to 8.26±9.46 (range, 0–48, median 5), after therapy (p-value<0.00).

Distribution of our homozygous, heterozygous, and compound heterozygous patients regarding the response to colchicine and disease severity score is shown in *Table 3*. Out of 95 patients:

**Table 3.** Homozygotes, and heterozygotes and compound heterozygotes regarding colchicine dose, response to therapy, and severity score.

**Variable**	**Homozygote (n=24)**	**Heterozygote (n=63)**	**Compound heterozygotes (n=8)**
Colchicine dose to control attacks mg/day (mean±SD, median)	1.53±0.35 (median 1.5)	1.34±0.42 (median 1)	1.25±0.46 (median 1.25)

*Response to therapy*			
Complete, n (%)	2 (8.3%)	18 (28.6%)	2 (25%)
Incomplete, n (%)	22 (91.7%)	44 (69.8%)	6 (75%)
No response, n (%)	0 (0.0%)	1 (1.6%)	0 (0%)

*Severity score*			
Mild, n (%)	0 (0.0%)	9 (14.3%)	1 (12.5%)
Moderate, n (%)	15 (62.5%)	40 (63.5%)	6 (75%)
Severe, n (%)	9 (37.5%)	14 (22.2%)	1 (12.5%)

- Complete response was noted in 22 (23.16%) patients, as follows: E148Q mutation in 9 patients, V726A mutation in 3 patients, M680I mutation in 3 patients, M694V mutation in 4 patients, and one patient per each of the following mutations M694I, M680I-V726A, and M680I-V726A.
- Incomplete response in 72 (75.79%) patients, as follows: E148Q mutation 15 patients, V726A mutation 15 patients, M680I mutation 16 patients, M694V mutation 13 patients, M694I mutation 6 patients, one patient per each of the following mutations M680I-V726A, M694I-E148Q, M694V-V726A, M680I-P369S, M680I-A744S, M694V-M694I, and M762V-M694V.
- No response in only one patient (1.05%) (V726A heterozygous mutation).

Of note, 20 out of 71 heterozygous/compound heterozygous patients (28.17%) had a complete response to therapy, that number was statistically significant than that reported among homozygotes who demonstrated a complete response to therapy as well (2 out of 24, 8.33%) (p-value =0.04).

Regarding severity of the disease, out of 95 patients:

- Mild scores were reported in 10 patients (10.53%) (E148Q mutations in 3 patients, M680I mutation in 3 patients, and one per each of the following mutations M694V, M694I, P369S, and M762V-M694V mutations),
- Moderate scores in 61 patients (64.21%) (E148Q mutation in 16 patients, V726A mutation in 14 patients, M680I mutation in 12 patients, M694V mutation in 7 patients, M694I mutation in 6 patients, M680IV726A in 2 patients, and one patient per each of the following mutations M694I-E148Q, M680I-P369S, M680I-A744S, and M694V-M694I mutations)
- Severe scores in 24 patients (25.26%) (E148Q mutation in 5 patients, V726A mutation in 5 patients, M680I mutation in 4 patients, M694V mutation in 9 patients, and M694V-V726A mutation in one patient).

**Figure 1.**
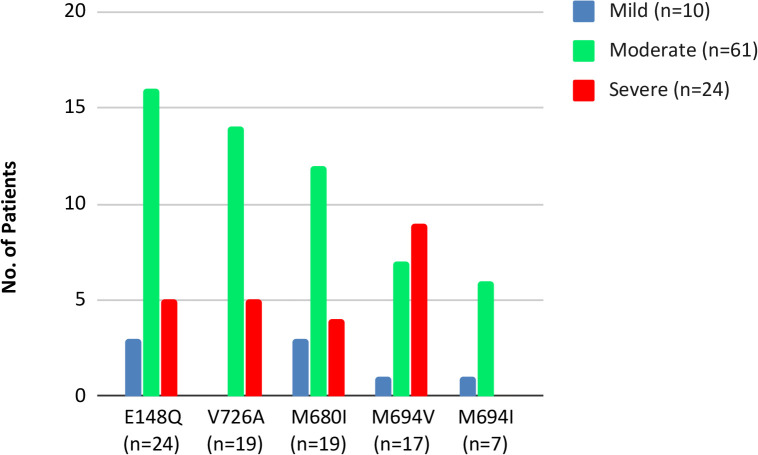
Most frequent genotypes and disease severity score.

The most common five mutations in our patients (E148Q, V726A, M680I, M694V, and M694I) were compared regarding demographic and clinical data, severity scores and response to colchicine (*Table 4*). Parental consanguinity and family history of FMF were reported in a higher percentage of patients with M694V mutations (41.18%). The mean age of onset for patients with M680I (4.44±2.25 years) was slightly lower than that of other mutations. As mentioned before, fever is a cardinal feature of presentation in FMF patients; however, it was reported in a lower percent of patients with M694I mutation (71.43%). Arthritis/arthralgia was reported in higher percent of patients with M694V, and E148Q (88.24%, and 87.50%, respectively). ELE was a common feature in V726A mutation (reported in 36.84% of patients with this mutation), and chest pain was a common feature in M694V mutation (reported in 41.18% of patients with this mutation). A better response to colchicine therapy was noted in patients with E148Q mutation, with 37.50% of patients with this mutation showed a complete response to therapy. According to disease severity score, more severe scores was detected in higher percent of patients with M694V (52.94%) (*Figure 1*); of note, the only compound heterozygote patient with M694V-V726A mutation had a severe disease score as well.

**Table 4.** Demographic and clinical data and response to colchicine in the most frequent mutations detected.

**Variable E148Q (n=24)**		Mutations^*^				

		**V726A (n=19)**	**M680I (n=19)**	**M694V (n=17)**	**M694I (n=7)**	
Male/female		12/12	9/10	9/10	7/10	5/2

Family history of FMF, n (%)		5 20.83%	3 15.79%	4 21.05%	7 41.18%	1 14.29%

Consanguinity, n (%)		9 37.5	3 15.79%	8 42.11%	11 64.71%	2 28.57

Age of onset years mean±SD median		4.65±2.63 4	5.16±2.41 5	4.44±2.25 4	4.73±2.38 4	6.51±2.66 5

*Clinical features (%)*						
Fever		95.83%	100%	100%	100%	71.43%
Abdominal pain		95.83%	94.74%	100%	94.12%	100%
Joint pain/arthritis		87.50%	78.95%	84.21%	88.24%	42.86%
Erysipelas-like erythema		16.67%	36.84%	15.79%	17.65%	0.00%
Chest pain		12.50%	15.79%	10.53%	41.18%	28.57%

Appendectomy, n (%)		3 12.50%	2 10.53%	1 5.26%	2 11.77%	1 14.29%

Response to colchicine	Complete, n (%)	9 (37.50%)	3 (15.79%)	3 (15.79%)	4 (23.53%)	1 (14.29%)

	Incomplete, n (%)	15 (62.50%)	15 (78.95%)	16 (84.21%)	13 (76.47%)	6 (85.71%)

	No response, n (%)	0 (0.00%)	1 (5.26%)	0 (0.00%)	0 (0.00%)	0 (0.00%)

* Patients with compound heterozygous genotypes were not included.

## DISCUSSION

FMF is one of the most frequent periodic febrile syndromes (12).^12^ Individual and ethnic variations in both frequency and clinical profile of FMF may be noted; the clinical manifestations of disease in the same patient may differ over time as well.^13,14^

Our study included 95 paediatric patients already diagnosed clinically with FMF and with positive-MEFV mutation; a slight female dominance was noted in our patients comes in agreement with Duşunsel et al.^15^ Studies reported FMF in both sexes in a similar ratio.^16^ It was noted that the mean age onset and the mean age at diagnosis in our patients were lower than those reported in other studies.^15,17^

Family history of FMF was reported in 22.11%, and consanguinity was detected in 37.89% of our patients. Different studies had reported family history and parental consanguinity in percentages that differ from our results. That observation could be related to the different familial predisposition of FMF in certain population.^18,19^

According to MEFV gene mutations analysis, more than 300 mutations have been reported^20^; the five most frequent mutations in the Middle East are M694V, M694I, V26A, E148Q, and M680I mutations.^7^ Our results show that the most frequently reported mutations were E148Q, V726A, M680I, M694V, and M694I and were detected in 25.26%, 20%, 20%, 17.89%, and 7.37% of FMF patients, respectively. Our results were some what similar to those reported by El-Shanti et al. with E148Q and V726A as the most frequent mutations followed by M694V mutation.^21^; On the other hand, our results were not similar to those noted by El-Garf et al.,^18^ who stated that V726A mutation was the most frequent genotype reported among their patients was noted in 41.2% of patients, followed by M694V, M680I, E148Q and M694I mutations in 32.4, 29.4, 25 and 20.6% of patients, respectively. Mutational heterogeneity of FMF could explain those differences among Egyptians. Our results were also different from other studies, because *MEFV* gene mutations differ in different ethnic’s studies with M694V, V726A, M680I, M694I, and E148Q mutations frequencies of 42.5%, 23.1%, 9.6%, 14.1%, and 10.7 5%, respectively among Arabs,^22^ 77%, 12.3%, 0.6%, 0%, and 10.2%, respectively among Jews,^23^ and 71.3%, 8.5%, 15%, 1.7%, and 3.5%, respectively among Turkish population.^24^ Different studies reported the most frequent MEFV mutations among their patients were M694V, followed by V726A, then M680I^25,26^; these mutations were in the fourth, second and third frequencies in our patients, respectively.

In our study, 25.26% of patients were homozygotes (M694V/M694V was the most frequent), 66.32% were heterozygotes (E148Q was the most frequent), and 8.42% were compound heterozygotes (M680I-V726A was the most frequent). It was expected to find a significant number of patients with family history of FMF and a lower mean age at diagnosis among homozygotes in comparison to heterozygotes and compound heterozygotes because of the recessive pattern of inheritance of FMF.

Fever was the most common manifestation among our patients and was reported in 96.84% of patients, followed by abdominal pain, arthritis, ELE and chest pain in 94.74%, 78.95%, 20%, and 17.89%, respectively. Manna et al.^27^ reported high fever and abdominal pain as the main presenting symptoms of FMF in 94%, and 83% of patients respectively. In Salehzadeh’s study,^26^ 88.1% of patients had fever, 93.3% had abdominal pain, 25.6% had arthralgia, and only 7.7% had arthritis. Ebrahimi-Fakhari et al.^28^ also reported fever, abdominal pain, arthritis, chest pain, and ELE in 78%, 95%, 59%, 32%, and 23% of patients respectively.

In our study, proteinuria was reported in 5.26% and was associated with E148Q, M694V, and M680I-P369S mutations. Amyloidosis was not reported according to renal biopsy in any of our patients. Salehzadeh^26^ also reported one case of amyloidosis among 403 patients included in the study, however, that case was associated with M694I-M694I mutations.

In our study, FMF was associated with other inflammatory conditions in 13.68% of patients; JIA, HSP, SLE, IBD were the associated disorders. M680I mutation was the most frequently associated mutation, followed by E148Q mutation, and then V726A and M694V mutations. MEFV mutation may result in upregulation of the innate immune system with increased IL-1β production and exaggerated initial response to the environmental triggers.^29^

To control the attacks, colchicine therapy was given to all our patients in a dose of 0.5–2 mg/day with a mean dose 1.38±0.41 mg/day; 23.16% showed complete response, 75.79% showed incomplete response, only one patient (1.05%) was unresponsive to therapy and that patient had V726A mutation. The mean colchicine dose to control attacks was significantly lower in heterozygotes/compound heterozygotes and with significantly better response to therapy than homozygotes. M694V homozygosity may be a clue for less colchicine response,^30^ 9 out of 24 homozygotes (37.50%) were M694V-M694V that could explain the less colchicine response observed in those patients. Although colchicine is effective, compliance with therapy is important for disease control and management.^31^ Compliance in our patients was not good, that may be another explanation to the incomplete or no response to therapy.

Parental consanguinity and family history of FMF were reported in higher percentages of patients with M694V mutations. The mean age of onset for patients with M680I was slightly lower than that of other mutations. Nearly similar lower age of onset with M680I mutation was observed by Özturk et al.^32^

Arthritis/arthralgia was less frequently reported in patients with M694I mutation and was reported in higher percentages in patients with M694V, and E148Q mutations. Arthritis accompanies M694V and E148Q mutations as reported by Kincir et al.^33^ ELE was observed in higher percent of patients with V726A and M694V mutations (36.84 and 17.65%, respectively). ELE is a pathognomonic feature in FMF with an incidence ranging from 3% to 46%, and is commonly associated with M694V mutation^(34)^.

Chest pain was a common feature in M694V mutation (41.18% of patients with this mutation); Özturk et al.^32^) also reported that pleurisy was a common feature with M694V mutations (75% of patients carrying this mutation). A better response to therapy was noted with E148Q mutation, and 37.50% of patients that had this mutation showed complete response. Poor compliance and/or intolerance to colchicine could be important factors for incomplete or no response to therapy that was observed in our patients. Disease severity score was detected in a higher percent of patients with M694V mutation (52.94%). MEFV mutations differ in correlation with disease severity; E148Q is recognized to have a milder clinical course,^35^ while M694V is associated with a more severe disease form and a higher risk of amyloidosis than other mutations.^36^ It is important to note that some authors considered E148Q as a functional polymorphism and not a disease-causing mutation.^37,38^ However, the role of E148Q mutation in the pathogenesis of FMF remains still unclear. Aydın et al. reported that E148Q as a disease-causing mutation; they noted that patients with such mutation could present with late-onset and milder disease course and well response to colchicine as compared with patients who carry other mutations.^39^

## CONCLUSION

E148Q, V726A, M680I, M694V and M694I mutation are the most frequent mutations among our patients with FMF denoting the heterogeneous mutation pattern and the milder form of the disease among Egyptian patients with FMF compared to other populations. ELE is not uncommon feature of the disease especially with V726A mutations. M694V mutations may indicate more severe disease scores with more prevalence of arthritis and pleurisy. Further studies on larger scales are needed to establish a good genotype-phenotype correlation among Egyptian FMF paediatric patients.
